# A dynamic early-warning method for bridge structural safety based on data reconstruction and depth prediction

**DOI:** 10.1371/journal.pone.0324816

**Published:** 2025-06-03

**Authors:** Yanqing Men, Hu Li, Fengzhou Liu, Yongliang Huang, Mingxin Gao, Xiaohui Wang, Hao Xie, Jianxin Cao

**Affiliations:** 1 Jinan Rail Transit Grp Co Ltd, Jinan, China; 2 Shandong Hi-speed Group Co Ltd, Jinan, China; 3 School of Oilu Transportation, Shandong University, Jinan, China; 4 School of Transportation Science and Engineering, Harbin Institute of Technology, Harbin, China; 5 Shandong Rail Transit Research Institute Co Ltd, Jinan, China,; 6 Jinan Rail Transit Urban Construction Segment Manufacturing Co Ltd, Jinan, China; University 20 Aout 1955 skikda, Algeria, ALGERIA

## Abstract

The structural response of bridges involves a complex interplay of various coupled effects, rendering the identification of long-term variation trends inherently challenging. Consequently, effectively detecting and alerting abnormal monitoring data for bridge structures under complex coupled loads remains a significant difficulty. To address this issue, this study proposes a dynamic early-warning method for bridge structural safety, leveraging data reconstruction and deep learning-based prediction. First, the singular value decomposition (SVD) algorithm is employed to decompose and reconstruct the monitoring data based on the contribution rate of influencing factors, thereby decoupling the data from various coupled effects. Second, a deep learning architecture utilizing a long short-term memory (LSTM) network is applied to establish a prediction model for each group of decomposed monitoring data, significantly enhancing prediction accuracy. Building on this foundation, the dynamic early-warning system for bridge structural safety is realized by integrating anomaly diagnosis theory with both predicted and measured data. A validation case using measured strain data demonstrates that the proposed method accurately predicts bridge strain data and calculates real-time adaptive thresholds, enabling real-time detection of anomalous monitoring data.

## 1. Introduction

In recent years, the scale of transportation infrastructure has expanded rapidly, leading to the construction of numerous bridges of varying types worldwide. Structural health monitoring (SHM) systems have been implemented on many significant large bridges to enable real-time monitoring of structural physical quantities, such as deformation and stress. Furthermore, advancements in wireless sensing, the Internet of Things (IoT), and optical fiber sensing technologies have facilitated the installation and application of SHM systems on small and medium-span bridges. However, in real-world operating environments, the structural response data collected by SHM systems are influenced by various complex factors, including ambient temperature fluctuations, random vehicle loads, and material shrinkage and creep [1–3]. The coupling effects of these factors significantly hinder the accuracy of early-warning systems for bridge structural safety, often resulting in missed or false alarms, which pose considerable challenges for bridge managers [4,5]. Consequently, developing methods to establish accurate early-warning thresholds based on real-time structural monitoring data remains a critical research focus and challenge in the field of structural health monitoring [6,7].

Relevant scholars have conducted in-depth research on safety diagnosis and early-warning systems for structural states using real-time monitoring data [8–14]. Fae Azhari et al. [15] introduced a probabilistic analysis-based early-warning framework for bridge erosion, which enables the prediction of potential erosion in advance. Fan et al. [16] employed the cointegration algorithm to derive a linear combination of two non-stationary structural response time series as a warning index. This approach generates a more stable cointegration residual series, facilitating the early detection of cable force anomalies and reducing false alarms. Zhu et al. [8] proposed a novel early-warning method for bridge structural states based on fuzzy comprehensive evaluation, which comprehensively assesses bridge safety using diverse real-time monitoring data. These methods typically establish multi-level warning thresholds based on benchmark models derived from historical monitoring data, thereby enabling the early-warning of structural states. However, early-warning systems relying on fixed threshold values exhibit significant limitations, as they fail to adequately account for the long-term effects of environmental and structural variability. If the threshold is set too high, the system may not issue alarms even when data anomalies occur, compromising the effectiveness of the warning. Conversely, if the threshold is too low, frequent alarms may result in unnecessary maintenance costs.

By leveraging historical monitoring data, a temporal correlation model can be established, serving as an effective early-warning mechanism for structural safety. This approach involves the development of a dynamic early-warning model capable of accurately predicting future monitoring data [17–19]. However, current prediction methodologies for structural monitoring data predominantly rely on multivariate nonlinear regression, machine learning, and similar techniques to model the relationship between structural responses and external factors such as environmental temperature and wind speed [20–27]. While these methods can forecast potential outcomes of monitoring data, they fall short in predicting future monitoring data with precision. Traditional machine learning approaches, including support vector regression, BP neural networks, and autoregressive moving average methods, exhibit limited accuracy in predicting future monitoring data and often fail to meet the requirements for dynamic early-warning systems [28–31]. Furthermore, the prediction of future monitoring data is further complicated by the various coupling effects influencing bridge structural monitoring data.

With the continuous advancement of computer hardware and the rapid evolution of big data analysis algorithms, in-depth research has been conducted on data prediction algorithms based on deep learning. Deep learning networks feature more complex deep architectures and hidden layers, and possess powerful nonlinear fitting capabilities as well as automatic feature extraction functions. Consequently, they are increasingly being applied in the big data processing for structural health monitoring [32–34]. Zhu et al. [35] proposed a deep learning-based method for an accelerometer-driven fatigue crack monitoring system. The proposed B-CNN classifier is further employed to predict crack conditions from upcoming measurements. Du et al. [36] put forward a heterogeneous structural response prediction framework grounded in the deep learning model. This approach can effectively explore the spatiotemporal correlations within structural monitoring data. It enables multi-step data prediction and enhances the accuracy of safety warnings. Shang et al. [37] utilized deep convolutional neural networks and denoising autoencoders to efficiently extract structural damage characteristics under the influence of time-varying temperatures. Based on this, structural damage warnings are issued through a moving average control chart. Rastin et al. [38] presented a damage detection framework relying on convolutional neural networks. The framework trains the stacked acceleration matrix and normalizes the diagnostic vectors of the base state and the unknown structural state into unit vectors. Then, the Euclidean distance of these diagnostic vectors is adopted as an early-warning indicator for structural safety. Wang [39] proposed a deep residual network framework for data prediction and anomaly detection in civil engineering structures. In a long-term operational environment, bridge structures are affected by various coupling effects. It is challenging to effectively uncover the deep temporal correlation patterns in the data merely by conducting time series modeling on the original monitoring data.

To solve the above problems, considering that the coupling effects of the same type of monitoring data within a bridge are similar, a dynamic early-warning method for bridge structure safety based on data reconstruction and depth prediction is proposed in this paper. The multi-dimensional monitoring data were decomposed and reconstructed according to the contribution rate of influencing factors. On this basis, the temporal association model is established for the decomposed data of each feature subspace by using a LSTM network, which can accurately predict prospective multi-step monitoring data. Then the dynamic warning threshold can be calculated, which not only improves the warning precision but also avoids the occurrence of false alarm to the greatest extent. Recent advancements in deep learning have spurred the development of diverse LSTM variants tailored for complex temporal modeling tasks. For instance, bidirectional LSTM (BiLSTM) architectures, which process sequences in both forward and backward directions, have proven effective in capturing contextual dependencies in vibration-based structural damage detection [40]. Hybrid frameworks integrating convolutional neural networks (CNNs) with LSTM, such as CNN-LSTM, leverage spatial feature extraction via CNNs followed by temporal modeling through LSTMs, demonstrating superior performance in structural response prediction under multi-source environmental influences [41]. Additionally, attention-enhanced LSTM models have been employed to prioritize critical temporal features in long-term monitoring data, improving anomaly detection accuracy [42]. While these advanced architectures offer notable benefits in specific contexts, the proposed method prioritizes computational efficiency and interpretability. By leveraging SVD to decouple strain data into subspaces dominated by distinct influencing factors (e.g., temperature, traffic loads), the temporal dynamics within each subspace are simplified, enabling a standard LSTM to effectively model unidirectional, long-range dependencies without requiring bidirectional or hybrid complexity. This streamlined approach aligns with studies demonstrating that optimized LSTM architectures, combined with robust preprocessing, achieve state-of-the-art performance in SHM applications [43], particularly where real-time deployment and resource constraints are critical considerations.

The remainder of this paper is arranged as follows. In Section 2: Methods,the algorithm theory of this paper is described in detail. In Section 3: Example with an actual bridge, the prediction accuracy of the proposed algorithm for future monitoring data is verified by using the strain monitoring data of actual bridge structures, and the accuracy is compared with that of traditional machine learning methods. In addition, the abnormal changes of strain caused by different degrees of structural damage are simulated by finite element model. The proposed method is used for dynamic early-warning of the abnormal condition, and the effectiveness of the proposed algorithm is verified. Finally, the conclusions are drawn in the section 4: Conclusions.

## 2. Methods

This section mainly describes the algorithm theory of the proposed method. First, the projection reconstruction algorithm of monitoring data based on singular value decomposition is proposed to realize the decomposition and reconstruction of monitoring data according to the contribution rate of influencing factors. Second, a prediction algorithm of prospective multi-step monitoring data based on a LSTM network is proposed. Finally, a dynamic early-warning method for structural safety based on predictive and measured data is proposed.

### 2.1. Decomposition and reconstruction of structural monitoring data

The SHM system of bridges generally requires the collection of long-term monitoring data, such as structural displacement and strain. The variations in these monitoring data serve as a comprehensive manifestation of various coupling effects. These effects mainly encompass structural temperature loads, wind loads, other external loads, as well as material shrinkage and creep, among others. The outcomes of directly predicting the monitoring data under the influence of coupling actions often exhibit substantial errors. Such errors are unable to meet the requirements of dynamic early-warning. Consequently, in this section, a projection reconstruction method for monitoring data based on singular value decomposition is proposed. This method is designed to decompose the monitoring data affected by the coupling effect to the greatest extent possible according to the contribution rate of influencing factors, thereby providing a foundation for enhancing the prediction accuracy of monitoring data.

The same type of multi-channel monitoring data is divided into training sets and test sets, as shown below.


$$X=[X_{tra},X_{test},,\mathrm{\backslash ]}$$ (1)

where, **X** is a sample set of multi-channel monitoring data; $X_{tra}\in {ℜ}_{n\times m_{1}}$ is the training set, and *n* denotes the number of sensors and *m*_1_ denotes the number of the samples of training set;$X_{test}\in {ℜ}_{n\times m_{2}}$ is the test set, and *m*_2_ denotes the number of the samples of test set.

The covariance matrix η of the training sample set of monitoring data is constructed, which is decomposed by SVD algorithm, as shown in Eq. (2).


$$\mathrm{\backslash [}\eta =\frac{1}{m_{1}-1}X_{tra}^{T}\cdot X_{tra}=USV^{T}\;,\mathrm{\backslash ]}$$ (2)

where **S** is a singular value matrix of the covariance matrix, which is a diagonal matrix. **U** and **V** are the left and right singular vector matrices of the covariance matrix, respectively. The singular value matrix **S** can be represented as follows.


$$\mathrm{\backslash [}S=\left[\begin{array}{cccc} {}*20cs_{1} & 0 & 0 & 0\\ 0 & s_{2} & 0 & 0\\ 0 & 0 & \ddots & 0\\ 0 & 0 & 0 & s_{n} \end{array}\right]\;,\mathrm{\backslash ]}$$ (3)

where $s_{1},s_{2},\cdots ,s_{n}$ are singular values and are arranged in descending order. The larger these values, the greater influence of the corresponding factors on the monitoring data. Calculate the cumulative contribution rate χ of the *k* ~ *n* singular values, as shown in Eq. (4).


$$\mathrm{\backslash [}\chi =\sum_{i=k}^{n}s_{i}^{2}/\sum_{i=1}^{n}s_{i}^{2}\;,\mathrm{\backslash ]}$$ (4)

where the value of k is determined according to the contribution rate, which generally needs to meet χ≤5\nonumber%.

The training set and verification set of monitoring data are projected to the previous each singular vector or matrix **U**_*k~n*_. Meanwhile, the each feature subspace obtained after projection is re-mapped to the original data space. Through the above process, the monitoring data can be decomposed according to the contribution rate of the influencing factors of SHM data, as shown in Eqs. (5) and (6).


$$\mathrm{\backslash [}\Theta_{tra,j}=U_{j}U_{j}^{T}X_{tra}\;,\mathrm{\backslash ]}$$ (5)

$$\mathrm{\backslash [}\Theta_{test,j}=U_{j}U_{j}^{T}X_{test}\;,\mathrm{\backslash ]}$$ (6)

where $U_{j}$ denotes anyone singular vector before the kth left singular vector or singular matrix Uk ~ n. $U_{j}^{T}X$ is the process of mapping the monitoring data samples to the feature subspace that reflects the information of main coupling load factors. $U_{j}U_{j}^{T}X$ is the process of remapping monitoring data samples from each feature subspace to the original data space. $\Theta_{tra,j}$ and $\Theta_{test,j}$ denote the decomposed monitoring data training set and test set, respectively.

### 2.2. Future multi-step data prediction based on a LSTM network

#### 2.2.1. Principles of LSTM network.

**Fig 1:**
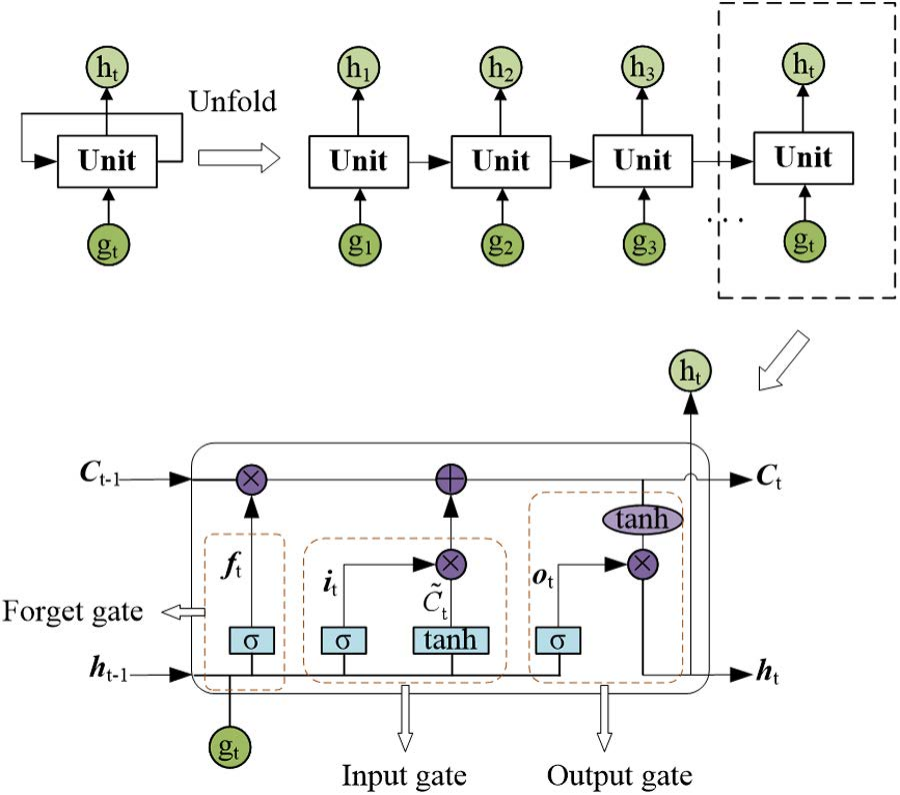
The structure diagram of a LSTM network.

The LSTM network is a type of recurrent neural network (RNN) with a distinctive structure. It has been extensively applied in various deep learning tasks, such as weather change prediction, structure lifetime prediction, and data timing association mining, among others [44,45]. The RNN is a deep learning model that primarily takes sequence data as its input. In a typical RNN, there are usually an input layer, a hidden layer, and an output layer. The hidden layer serves as the core of the recurrent process and is composed of multiple deep units. The output value of each unit is determined by both the input to that unit and the output of the preceding units. However, in traditional RNNs, it is challenging for data information to propagate from earlier units to subsequent units. As a result, RNNs suffer from the “long dependency” problem [44]. The fundamental distinction between an LSTM network and traditional RNNs lies in the fact that the LSTM network is capable of capturing the temporal correlations within data sequences. In the LSTM network, a specialized unit architecture is employed to replace the conventional unit in the RNN’s hidden layer. This specialized architecture enables the learning of long-term data dependencies, and its structure is illustrated in Fig 1.

The unit architecture of LSTM network is more complex, including the forget gate, the input gate, the output gate and the state vector connecting all the units in series. The state vector runs along the entire chain of network structures. Since it consists of only linear dot multiplication or addition operations, it is easy for information to flow unchanged through the chain. The calculation process of the forgot gate can be implemented through the Sigmoid layer, which extracts the output ***h***_*t*-1_ of the previous cell and the input ***g***_*t*_ of the current cell, and outputs a number between 0–1 for each digit in the cell state vector ***C***_*t*-1_. The number 1 indicates that the information is completely retained, the number 0 indicates that the information is completely deleted, as shown in the Eq. (7).


$$\mathrm{\backslash [}f_{t}=\sigma \left(W_{f}\cdot \left[h_{t-1},g_{t}\right]+b_{f}\right)\;,\mathrm{\backslash ]}$$ (7)

where σ(·) denotes the Sigmoid function; ***f***_*t*_ is the activation vector of the forget gate at time *t*; ***W***_*f*_ and ***b***_*f*_ are the model parameter of the inpu*t* vector and the hidden layer vector in the forget gate, respectively.

The input gate gets the new information added to the state vector. First, the Sigmoid layer calculates what information will be updated. Second, the tanh layer creates a new candidate memory vector; Finally, the input gate multiplies them and passes this information to the state vector, as shown in Eqs. (8) and (9).


$$\mathrm{\backslash [}i_{t}=\sigma \left(W_{i}\cdot \left[h_{t-1},g_{t}\right]+b_{i}\right)\;,\mathrm{\backslash ]}$$ (8)

$$\mathrm{\backslash [}{\tilde{C}}_{t}=\tanh\left(W_{C}\cdot \left[h_{t-1},g_{t}\right]+b_{C}\right)\;,\mathrm{\backslash ]}$$ (9)

where tanh(·) denotes tanh function; ***i***_*t*_ is an activation vector of the input gate at time *t*; ***W***_*i*_ and ***b***_*f*_ are the model parameter of the inpu*t* vector and the hidden layer vector in the input gate, respectively. ${\tilde{C}}_{t}$ is the candidate memory vector at time t; ***W***_*C*_ and ***b***_*C*_ are the model parameter of the input vector and the hidden layer vector in the memory unit, respectively.

On this basis, the state vector output by the previous unit is multiplied with the activation vector generated by the forget gate of this unit, and the unwanted information is discarded. Meanwhile, by adding the input gate information to obtain useful information of this unit, a new state vector can be obtained as shown in the Eq. (10).


$$\mathrm{\backslash [}C_{t}=f_{t}\cdot C_{t-1}+i_{t}\cdot {\tilde{C}}_{t}\;,\mathrm{\backslash ]}$$ (10)

The output gate can obtain the output vector of the hidden layer. First, the output parts of ***h***_*t*-1_ and ***g***_*t*_ are calculated by the Sigmoid function. Second, the tanh function is used to process the state vector and multiply it with the output of the Sigmoid gate. Finally, the output vector is obtained, as shown in Eqs. (11) and (12).


$$\mathrm{\backslash [}o_{t}=\sigma \left(W_{o}\cdot \left[h_{t-1},g_{t}\right]+b_{o}\right)\;,\mathrm{\backslash ]}$$ (11)

$$\mathrm{\backslash [}h_{t}=o_{t}\cdot \tanh\left(C_{t}\right)\;,\mathrm{\backslash ]}$$ (12)

where ***i***_o_ is the activation vector of the output gate at time t. ***W***_o_ and ***b***_o_ are the model parameter of the output vector and the hidden layer vector in the output gate, respectively. ***h***_*t*_ is the hidden layer vector.

#### 2.2.2. Establishment of prediction model for prospective multi-step monitoring data.

Compared with the original coupling data, the decomposed monitoring data can be better mined for its long-term change rules. In this section, the LSTM network is used to construct a prospective multi-step prediction model for the decomposed and reconstructed monitoring data. Before modeling, each group of decomposed monitoring data is standardized to improve the precision of in-depth training, as shown in Eqs. (13) and (14).


\[Θ^tra,jc1=[Θtra,jc1−E(Θtra,j)]/[Θtra,jc1−E(Θtra,j)]D(Θtra,j)\nulldelimiterspaceD(Θtra,j)c1=1,2,⋯,m1,\] (13)

\[Θ^test,jc2=[Θtest,jc2−E(Θtra,j)]/[Θtest,jc2−E(Θtra,j)]D(Θtra,j)\nulldelimiterspaceD(Θtra,j)c2=1,2,⋯,m2,\] (14)

where ${\hat{\Theta }}_{tra,j}^{c_{1}}$ denotes the value of the ***c***_1_th sampling data from the jth trained data space after standardized processing; ${\hat{\Theta }}_{test,j}^{c_{2}}$ denotes the value of the ***c***_2_th sampling data from the *j*th test data space after standardized processing; E(·) and D(·) denote the mean and variance of a vector, respectively.

Set the sampling step length *g* for a single input and the prediction step length *f* for a single input. The data sliding window is used to store the training data into the input sample set and the output sample set, as shown in Eqs. (15) and (16).


$$\mathrm{\backslash [}{\hat{\Psi }}_{tra,j}^{input}\left\{h_{1}\right\}={\hat{\Theta }}_{tra,j}^{1+f\times \left(h_{1}-1\right)\backslash \;g+f\times \left(h_{1}-1\right)}h_{1}=1,2,\cdots ,\left(m_{1}-g-f\right)/g+1\;,\mathrm{\backslash ]}$$ (15)

$$\mathrm{\backslash [}{\hat{\Psi }}_{tra,j}^{output}\left\{h_{1}\right\}={\hat{\Theta }}_{tra,j}^{1+f\times \left(h_{1}-1\right)+g\backslash \;g+f\times h_{1}}h_{1}=1,2,\cdots ,\left(m_{1}-g-f\right)/g+1\;,\mathrm{\backslash ]}$$ (16)

where ${\hat{\Psi }}_{tra,j}^{input}\left\{h_{1}\right\}$ denotes the *h*_1_th input array from the *j*th trained data space; ${\hat{\Psi }}_{tra,j}^{output}\left\{h_{1}\right\}$ denotes the *h*_1_th output array from the *j*th trained data space.

Deep learning network architecture is designed. The sequence of architecture layers is: Time sequence input layer, LSTM network layer, Full connection layer, Regression output layer. The network is trained until the loss function is convergent, as shown in Eq. (17).


$$\mathrm{\backslash [}\Gamma \left(\theta \right)=\underset{\Theta }{\mathrm{\backslash arg}\min}{\left\|K_{j}\left({\hat{\Psi }}_{tra,j}^{input}\right)-{\hat{\Psi }}_{tra,j}^{output}\right\|}_{2}^{2}\;,\mathrm{\backslash ]}$$ (17)

where Γ(θ) is the loss function; θ is the hidden layer parameter set of the deep learning network; $K_{j}\left(\cdot \right)$ is the prediction function of prospective monitoring data for the *j*th data space, which is built through deep training.

According to the input step *g* and the prediction step *f*, the data sliding window is used to store the test data into the input sample set, as shown in Eq. (18).


$$\mathrm{\backslash [}{\hat{\Psi }}_{test,j}^{input}\left\{h_{2}\right\}={\hat{\Theta }}_{test,j}^{1+f\times \left(h_{2}-1\right)\backslash \;g+f\times \left(h_{2}-1\right)}h_{2}=1,2,\cdots ,\left(m_{2}-g-f\right)/g+1\;,\mathrm{\backslash ]}$$ (18)

where ${\hat{\Psi }}_{test,j}^{input}\left\{h_{2}\right\}$ denotes the *h*_2_th input array from the *j*th test data space. Based on the multiple trained deep learning networks, the input sample set of test data is used to predict the decomposed and reconstructed monitoring data, as shown in Eq. (19).


$$\mathrm{\backslash [}{\hat{\Psi }}_{test,j}^{pre}=K_{j}\left({\hat{\Psi }}_{test,j}^{input}\right)\;,\mathrm{\backslash ]}$$ (19)

where ${\hat{\Psi }}_{test,j}^{pre}$ denotes the prediction result of the prospective monitoring data for the *j*th data space. The prediction data are de-standardized and recombined to obtain the prospective multi-step prediction data $\Theta_{test}^{pre}$ for the structural response of bridges, as shown in Eq. (20).


$$\mathrm{\backslash [}\Theta_{test}^{pre}=\Xi \left({\hat{\Psi }}_{test,1}^{pre}+{\hat{\Psi }}_{test,2}^{pre}+\cdots +{\hat{\Psi }}_{test,j}^{pre}+\cdots +{\hat{\Psi }}_{test,k\backslash \;n}^{pre}\right)\;,\mathrm{\backslash ]}$$ (20)

where Ξ(·) denotes a de-standardized function.

### 2.3. Structural safety dynamic early-warning based on predictive and measured data

The prospective multi-step prediction data can be obtained by forecasting and recombining the decomposed and reconstructed monitoring data. On this basis, the dynamic early-warning for structural safety based on predictive and measured data can be further completed. The residual vector $\delta_{h}$ between the predictive and measured data can be calculated by using the verification data set under the health state of bridges, as shown in Eq. (21).


$$\mathrm{\backslash [}\delta_{h}=\Theta_{h}^{pre}-\Theta_{h}^{mea}\;,\mathrm{\backslash ]}$$ (21)

where $\Theta_{h}^{pre}$ is the predicted data in the health state. $\Theta_{h}^{mea}$ is the mean vector of the measured data in the healthy state. According to the anomaly diagnosis theory, the threshold vector can be defined, as shown in Eq. (22).


$$\mathrm{\backslash [}\{\begin{array}{c} {}*20c\gamma^{\text{up}}=\Theta_{d}^{pre}+\partial \cdot \Gamma_{0.95}\left(\delta_{h}\right)\\ \gamma^{\text{down}}=\Theta_{d}^{pre}-\partial \cdot \Gamma_{0.95}\left(\delta_{h}\right) \end{array}\;,\mathrm{\backslash ]}$$ (22)

where $\Theta_{d}^{pre}$ is the predicted data in the state to be diagnosed; $\Gamma_{0.95}\left(\cdot \right)$ is the 95% confidence rate function of a data set; $\gamma^{up}$ and $\gamma^{down}$ are the upper and lower limits of the threshold vector, respectively. ∂ is a guarantee coefficient of dynamic early-warning, which is generally 1.1 to 1.2.

**Fig 2:**
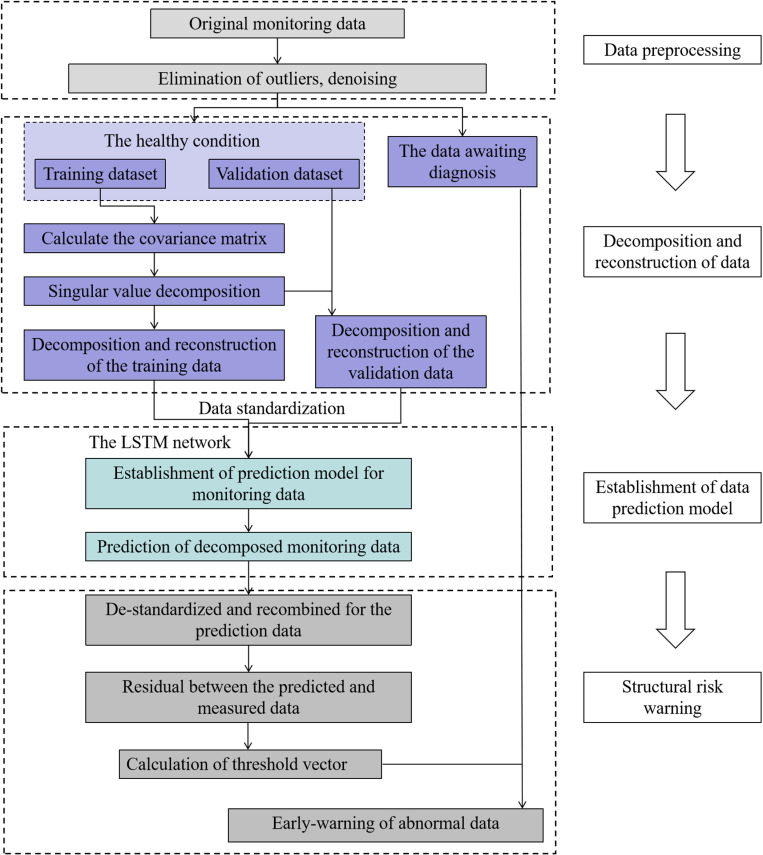
Overall framework of the proposed method.

The overall flowchart of the proposed method is shown in Fig 2.

## 3. Example with an actual bridge

### 3.1. Description of the SHM system of an actual bridge

**Fig 3:**
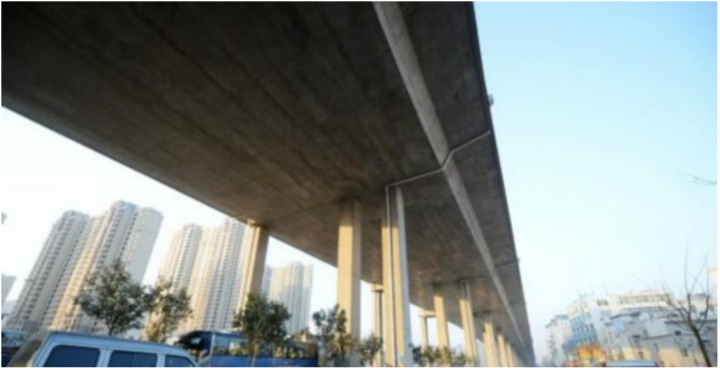
Photograph of the actual bridge.

**Fig 4:**
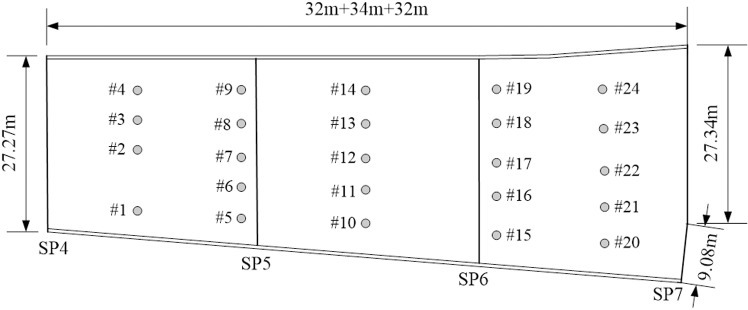
Placement and number of the strain sensors.

**Fig 5:**
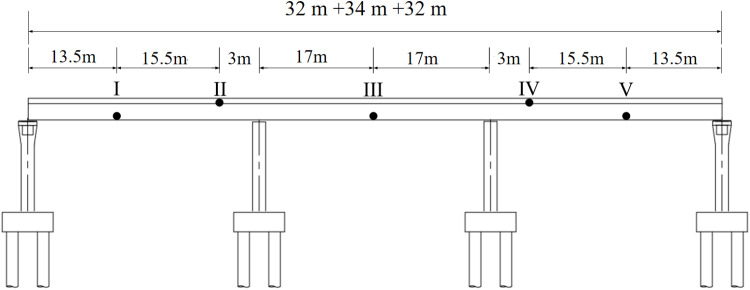
Strain monitoring section.

**Fig 6:**
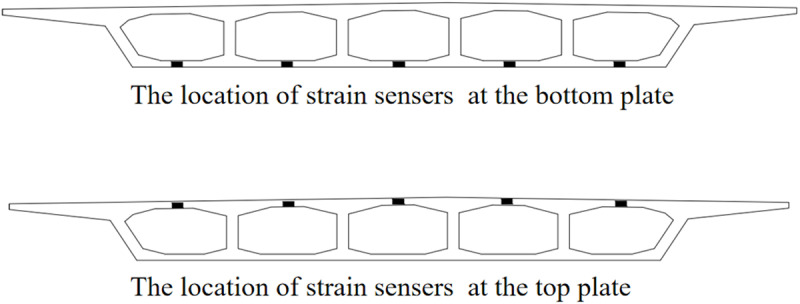
The lateral distribution position of the strain sensors.

In this paper, the strain monitoring data of a continuous beam bridge are used to test the accuracy of the proposed dynamic warning method. The bridge is a 3 × 30m single-box five-room continuous beam bridge, as shown in Fig 3. The SHM system was installed during the construction period. A total of 24 strain monitoring sensors are arranged in 5 critical structural sections of the bridge, as shown in Fig 4. The strain sensors are arranged on the bridge bottom plate at the maximum positive bending moment section (sensor number: #1 ~ #4, #10 ~ #14, #20 ~ #24), and the strain sensors at the fulcrum section are arranged on the bridge roof (sensor number: #5 ~ #9, #15 ~ #19), as shown in Fig 5 and Fig 6. The transverse stiffness of the middle part of the main beam box is small, which is easy to produce longitudinal cracks, structural reinforcement, water seepage and other diseases, resulting in the decline of structural stiffness. The strain sensors are arranged along the longitudinal bridge to monitor the longitudinal strain of the bridge. All strain sensors collect data synchronously every 10 minutes. The effectiveness of the proposed method is verified by using the long-term strain monitoring data.

**Fig 7:**
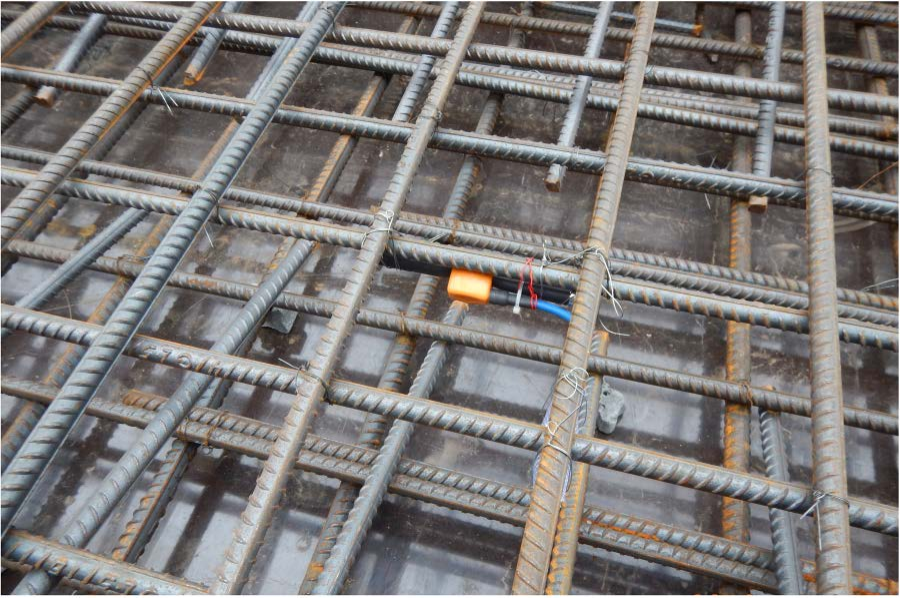
Installation of strain sensors during construction.

**Fig 8:**
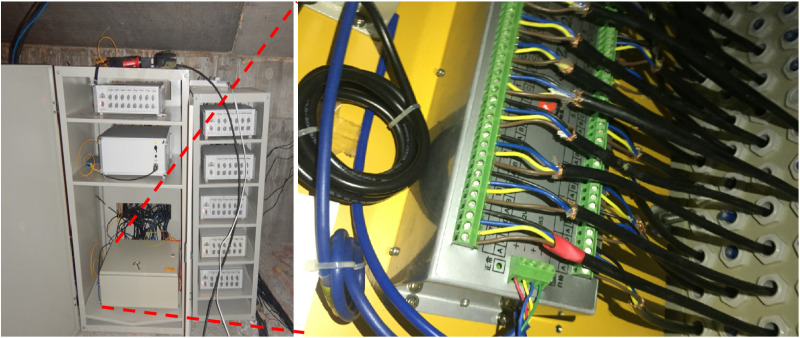
Simultaneous collection of strain monitoring data.

The sensors employed in this study were embedded intelligent vibrating wire strain gauges (Model JMZX-215HAT) manufactured by Changsha Jinma Measurement and Control Technology Co., Ltd., with their sensing parameters detailed in Table 1. The data acquisition system utilized a comprehensive acquisition and expansion module developed by the same manufacturer. The data acquisition system is equipped with multiple expansion modules for simultaneous measurement of 24-channel sensor data. The installation and acquisition of strain sensors are shown in Fig 7 and Fig 8.

**Table 1:** Sensor parameter.

Sensor performance	Parameter value	Sensor performance	Parameter value
Range	±1500 με	Temperature measurement accuracy	± 0.5 °C
Sensitivity	0.1 με	Scale distance	146 mm
Precision	0.5% FS	Dimension	24 mm × 37 mm × 150 mm
Temperature range	−40 °C~+120 °C	Expansion coefficient of steel string	12.2 με/°C

### 3.2. Establishment of a prediction model of future multi-step monitoring data

**Fig 9:**
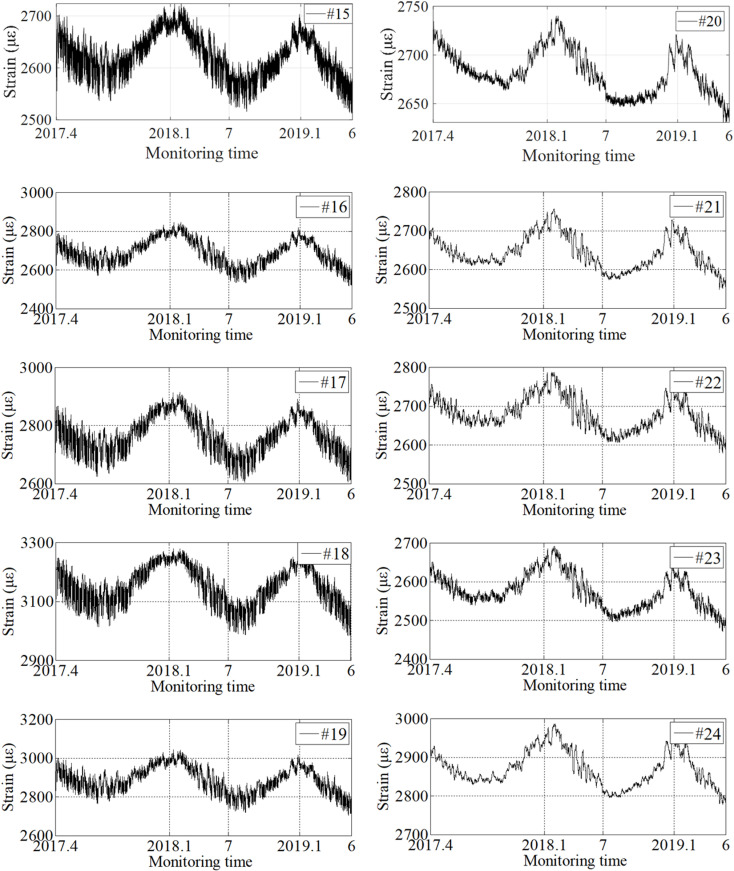
The original strain monitoring data (Measurement point #15 −19, Measurement point #20 −24).

**Fig 10:**
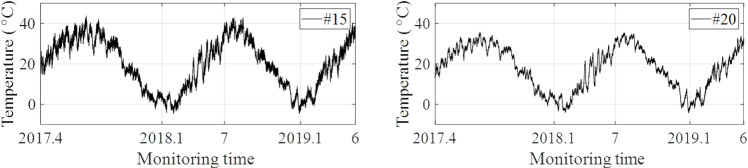
The temperature monitoring data (Measurement point #15 and #20).

In this section, strain monitoring data at the maximum positive bending moment cross section (#20 ~ #24) and the maximum negative bending moment cross section (#15 ~ #19) are selected respectively to establish two prediction models for future multi-step monitoring data. The bridge SHM system officially collects strain data after the bridge is put into operation. The bridge operation environment. The long-term variation trend of the original strain is shown in Fig 9. The ordinate represents the original reading of the strain sensor, which is independent of the strain change in the bridge structure. As the strain change is a relative quantity, the initial data collected serves as the strain reference number, and the difference between the subsequent data collected and the initial data is the strain change generated by the bridge. The long-term variation trend of temperature is shown in Fig 10. By comparing the strain and temperature data, it can be concluded that the annual trend of temperature change is basically the same. The influence on strain monitoring data is more complex, which not only shows the annual periodicity with temperature change, but also is affected by long-term traffic flow change, structural shrinkage and creep, et al. Therefore, on the one hand, it is difficult to directly define the early-warning threshold for structural safety based on long-term monitoring data. On the other hand, because the monitoring data is affected by various coupling effects, it is difficult to accurately obtain the change rule of the data when machine learning or deep learning algorithms are directly used for prediction, and thus the accuracy of early-warning cannot be guaranteed. Furthermore, the long-term strain variation trends of the bridge roof or floor are basically the same. Therefore, the proposed projection reconstruction method based on singular value decomposition will be effective to decompose the monitoring data under the coupling effect according to the contribution rate of influencing factors to the maximum extent, so as to provide a basis for improving the prediction accuracy of monitoring data.

Because of the long sampling interval of strain monitoring data, the number and location distribution of vehicles on the bridge will be different at each sampling time, so the vehicle load also exhibits strong randomness. Therefore, a cubical smoothing algorithm with five-point approximation [46] is used to reduce the influence of noise and random vehicle loads on strain monitoring data. In addition, to standardize the magnitudes of the changes in the strain monitoring data from the tops and bottoms of bridges and improve the predictive ability of the deep learning model, it is necessary to standardize the input and output data of the deep learning model before modeling.

**Fig 11:**
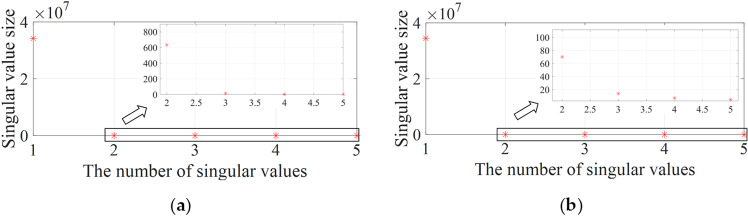
Singular value decomposition results. **(a)** Monitoring points #15 ~ #19 on the roof plate of bridge. **(b)** Monitoring points #20 ~ #24 on the bottom plate of bridge.

The monitoring data of two groups of strain sensors, #20 ~ #24 and #15 ~ #19, were selected to test the accuracy of the proposed method. A total of 10,000 monitoring samples in two months were selected as a training data set, and the following 3,000 monitoring samples were selected as the verification data set. The singular value decomposition algorithm was used to decompose the covariance matrix of the training data set. The sizes of the five singular values after decomposition were shown in Fig 11.

According to the size of all singular values, there are two main factors affecting the strain monitoring data. Therefore, it is necessary to map the monitoring data sample to three feature subspaces that reflect the information of the first, second and subsequent influencing factors. On this basis, the monitoring data samples are re-mapped from each feature subspace to the original data space according to Eqs. (5) and (6). The monitoring data after decomposition and reconstruction is normalized. Input sequence length and prediction steps were set to 200 and 20, respectively, to align with the temporal resolution of strain data (10-minute intervals) and ensure sufficient historical context for multi-step forecasting. The value of single input step is set to 200, and the value of single prediction step is set to 20. Data sliding window is used to store the data as input sample set and output sample set of training data.

In addition, it is necessary to establish a deep learning network architecture, which consists of sequence input layer, LSTM network layer, full connection layer, and regression output layer. The number of hidden layers is often related to the scale and complexity of the dataset. Adding more layers allows the network to better learn the features of the data, but excessive layers may result in overfitting issues. Generally, it is recommended to start with a small number of hidden layers and gradually increase them until satisfactory performance is achieved. In this study, the number of hidden layers of the LSTM network is set to 200. This configuration was determined through iterative experimentation, starting with fewer units (e.g., 50–100) and incrementally increasing complexity until validation loss plateaued. A deeper architecture (multiple LSTM layers) was initially tested but discarded due to overfitting risks and marginal accuracy gains. The Adaptive moment estimator (Adam) optimizer is selected as the network solver, which is suitable for deep learning of big data and can adjust the learning rate adaptively during model training. The small-batch gradient descent method is used to improve the running speed of the algorithm and ensure the convergence accuracy.

The determination of hyper-parameters is of particular importance. An initial learning rate of 0.005 was selected based on grid search trials (0.001–0.01). A step decay strategy was applied at epoch 25, reducing the learning rate by 20% to stabilize convergence during later training phases. A mini-batch size of 64 was chosen to balance memory constraints and gradient estimation stability. Dropout (rate = 0.2) was incorporated after the LSTM layer to mitigate overfitting, validated via cross-validation on the training set. A gradient threshold of 1 was enforced to prevent exploding gradients, particularly critical for long input sequences. Training was capped at 50 epochs, with monitoring of validation loss to halt training if no improvement occurred for 10 consecutive epochs. Root Mean Squared Error (RMSE) was used to penalize deviations between predicted and measured strain values, ensuring alignment with the regression objective. A held-out validation set (20% of training data) guided hyperparameter adjustments, ensuring generalization before final evaluation on the test set.

After initial training, sensitivity analyses were conducted to assess the impact of key parameters (e.g., hidden units, sequence length). For instance, increasing hidden units beyond 200 marginally improved accuracy but escalated computational costs disproportionately. Similarly, shorter input sequences (g < 150) degraded prediction stability due to insufficient historical context. These findings reinforced the final configuration’s suitability for balancing accuracy and efficiency in bridge SHM applications.

**Fig 12:**
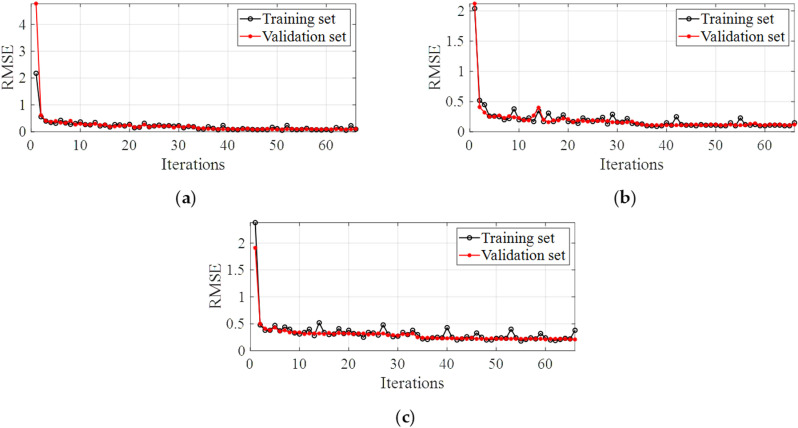
The convergent process of model loss function. **(a)** The first feature data space. **(b)** The second feature data space. **(c)** The third feature data space.

Taking measuring points #15 ~ #19 as an example, the convergence process of loss function of future multi-step monitoring data prediction model in training is shown in Fig 12. During repeated training, when the value of the loss function drops significantly and enters a steady trend period, it indicates that the trained network has reached a certain regression accuracy. By comparing the process of loss function convergence between validation set and training set, the proposed deep learning architecture has less probability of overfitting and good generalization ability.

### 3.3. Comparison and analysis of prediction model accuracy

**Fig 13:**
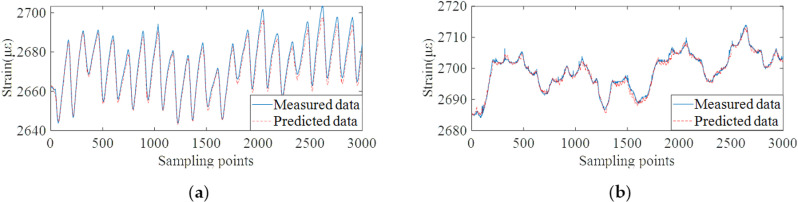
Data prediction results of the proposed method. **(a)** Monitoring points #15 on the roof plate of bridge. **(b)** Monitoring points #20 on the bottom plate of bridge.

**Fig 14:**
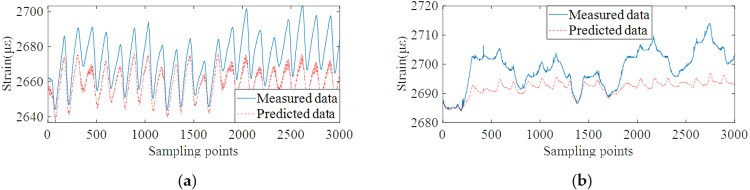
Data prediction results of SARIMA algorithm. **(a)** Monitoring points #15 ~ #19 on the roof plate of bridge. **(b)** Monitoring points #20 on the bottom plate of bridge.

**Fig 15:**
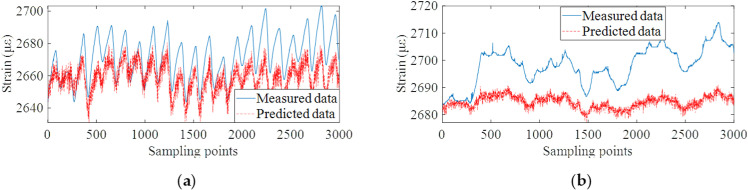
Data prediction results of SVM algorithm. **(a)** Monitoring points #15 ~ #19 on the roof plate of bridge. **(b)** Monitoring points #20 on the bottom plate of bridge.

Based on the well-trained data prediction model, the validation data set is used to test the prediction ability of the model. Different from the conventional method which establishes the mapping relationship between load and structural response, the proposed method can predict the future multi-step monitoring data, and then calculate the future multi-step dynamic warning threshold in advance. On this basis, the proposed algorithm was compared with the Seasonal autoregressive integrated moving average model (SARIMA) [47], and the relevant input and prediction step size were consistent with the proposed method. Autoregressive integrated moving average (ARIMA) algorithm is a common method for correlation modeling of time series data. Based on the ARIMA model, SARIMA introduces the ability of periodic cycle identification, while retaining the feature that ARIMA does not require data stationarity. The monitoring data prediction results of the proposed method and two comparison methods are shown in Figs 13–15.

From the results of Figs 13–15, it can be concluded that the proposed method can accurately predict future monitoring data by decomposing and reconstructing the original monitoring data and deeply mining the temporal correlation of monitoring data. The result of data prediction is obviously better than the other two methods, which can provide higher accuracy for the subsequent dynamic early-warning. The prediction results of SVM algorithm have the condition of overall data deviation. The reason is that the strain monitoring data are affected by many coupling factors. Meanwhile, the strain monitoring data contains the long-term shrinkage and creep trend and the change trend of traffic flow, which can not be effectively predicted by temperature load alone. The prediction accuracy of SARIMA algorithm for future multi-step data is much lower than that of the proposed algorithm. Table 2 shows the root-mean-square errors (RMSEs) of the three models for the predicted and measured data of all strain measuring points #15 ~ #19 and #20 ~ #24. The RMSEs of the strain prediction data and the measured data are within 3με.

**Table 2:** The RMSEs of the predicted and measured strain values.

Monitoring point	RMSEs after normalization (με)			Monitoring point	RMSEs after normalization (με)		
	The Proposed method	SARIMA	SVM		The Proposed method	SARIMA	SVM
#15	2.6869	14.8038	12.9804	#20	0.7378	8.1109	14.3337
#16	1.4602	13.0370	4.9376	#21	0.6265	7.1233	10.8026
#17	1.8621	12.6879	5.3345	#22	1.3222	7.0804	10.4728
#18	0.9066	12.1455	7.9249	#23	0.7276	7.2883	11.8362
#19	1.9501	13.6884	6.8082	#24	0.8044	8.3925	13.3346

### 3.4. Establishment of a prediction model of future multi-step monitoring data

**Fig 16:**
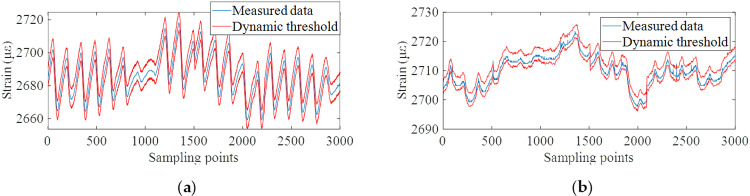
Dynamic early-warning results. **(a)** Monitoring points #15 ~ #19 on the roof plate of bridge. **(b)** Monitoring points #20 on the bottom plate of bridge.

Strain monitoring data of the verification set in the previous section are used as reference data, and the subsequent 3000 data sets are used as test data to be diagnosed. Based on the established data prediction model, the early-warning threshold vector can be calculated by accurately predicting the future multi-step monitoring data in the diagnosis state, and then the dynamic early-warning for abnormal monitoring data can be realized. Through calculation, the strain monitoring data of all monitoring points of the bridge did not exceed the warning threshold. Among them, the warning results of monitoring points #15 and #20 are shown in Fig 16. Since the construction time of the bridge is short, some common structural damage such as cracks and exposed reinforcement was not found through manual survey, which is consistent with the diagnosis results.

To verify the early-warning capability of the proposed method, the simulated strain changes caused by structural damage are added to the strain data of the test data set at measuring points #15 and #20 [9], as shown in the Eq. (23).


\[⌢\] (23)

where mvnrnd(μ,σ) denotes a generator for random number whose mean is μ and variance is σ; ${\frac{\mathrm{\backslash buildrel}\mathrm{\backslash lower}3pt\text{\textbackslash{}scriptscriptstyle\textbackslash{}frown}}{\varepsilon }}_{k,test}^{i}$ is the strain value after adding the simulated damage at the *k*th sampling value of the *i*th monitoring point in the test set.

In this section, a numerical example is used to investigate the deviation of strain monitoring data caused by structural damage. Through these numerical examples, the strain changes caused by different damage degrees can be obtained. In numerical examples, finite element models (FEMs) of three common types of bridge structures are established based on the actual bridge dimensions, which are continuous beam bridge with a single box and three chambers, continuous small box girder bridge and simply supported hollow slab bridge. The structural damage is set at the cross section of the maximum positive bending moment and the maximum negative bending moment, and local structural damage often occurs in these parts, such as concrete cracks, concrete spalling, wet joint damage, etc. Meanwhile, strain sensors are usually installed in these locations.

**Fig 17:**
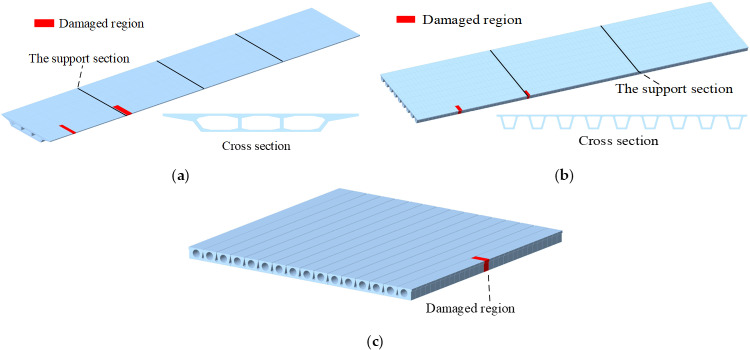
Damage locations of bridge structures. **(a)** Continuous beam bridge with a single box and three chambers. **(b)** Continuous small box girder bridge. **(c)** Simply supported hollow slab bridge.

The dimensions of the three types of bridge FEMs are shown in Table 3, and the damage location is shown in Fig 17. In the FEM, only the structural dead-weight and bridge deck pavement load are added. The bulk weight of the concrete is 25kN/m3 and the elastic modulus is 3.45 × 107kN/m^2^. In addition, the bridge deck pavement is converted into a uniform load added to the beam element. The beam element is used to simulate the main beam structure, which is also the most common finite element modeling method. In the three finite element models, the length of each longitudinal beam element is set to 0.5m. For the actual bridges, the common structural diseases lie in some local damage, e.g., the crack of concrete, the concrete spalling of superstructure of bridge, the wet joint damage etc. These damage always directly relate with the loss of stiffness of structures. Therefore, the stiffness loss of a single beam element is used to simulate structural damage [48–50]. Strain perturbation caused by structural damage is shown in Table 4. The results show that when 5% damage occurs in structural element, the strain change of bridge 1# is more than 5με, and that of the other two bridges is more than 10με. When 10% damage occurs in structural element, the strain change of bridge 1# is about 15με, and the strain change of the other two bridges is in the range of 20με ~ 30με.

**Table 3:** Size of FEMs of bridges.

The number of bridges	Bridge type	Bridge span	Bridge width
1#	Continuous beam bridge with a single box and three chambers	4 × 30 m	14.9 m
2#	Continuous small box girder bridge	3 × 30 m	23 m
3#	Simply supported hollow slab bridge	15.5 m	16 m

**Table 4:** Strain perturbation caused by structural damage.

Bridge	Damage location	Damage degree	Strain (με)/ Strain change (με)	
			Damaged element	Adjacent element
#1	The cross section with maximum positive bending moment	Health	137.0/ —	132.7/ —
		5%	143.5/ 6.5	132.2/ −0.5
		10%	150.6/ 13.6	131.6/ −1.1
	The cross section with maximum negative bending moment	Health	−169.3/ —	−103.7/ —
		5%	−176.4/ −7.1	−102.4/ 1.3
		10%	−184.1/ −14.8	−101.0/ 2.7
#2	The cross section with maximum positive bending moment	Health	243.3/ —	243.2/ —
		5%	255.7/ 12.4	242.9/ −0.3
		10%	269.5/ 26.2	242.4/ −0.8
	The cross section with maximum negative bending moment	Health	−264.5/ —	234/ —
		5%	−277.7/ 13.2	233.3/ −0.7
		10%	−292.3/ 27.8	232.6/ −1.4
#3	The cross section with maximum positive bending moment	Health	188.9/ —	188.7/ —
		5%	198.2/ 9.3	188.1/ −0.6
		10%	208.5/ 19.6	187.5/ −1.2

According to the strain deviation results of the above three typical bridges under different damage degrees, the simulated damage with an average value of 10με and variance of 0.01 is added to the last 1500 strain data from the #20 monitoring point of actual bridge. The simulated damage can reflect the deviation of strain monitoring data caused by real damage of bridge to a certain extent. Therefore, the method can not only use the measured data containing various real coupling effects, but also reflect the local structural damage.

**Fig 18:**
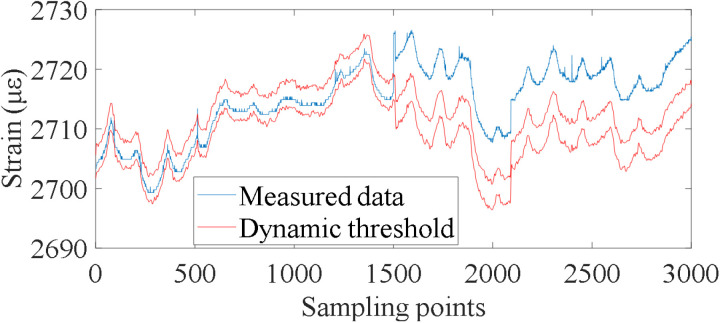
Dynamic early-warning results after the addition of simulated damage.

After adding simulated damage, the early-warning result of strain monitoring data at monitoring point #20 is shown in Fig 18. The early-warning result shows that the proposed method can effectively define the dynamic early-warning threshold and accurately warn the abnormal monitoring data caused by the damage by predicting the future multi-step monitoring data. Compared with the traditional method of setting fixed threshold, it can improve the early warning response sensitivity on the one hand, and effectively avoid the phenomenon of false early warning on the other hand. Therefore, the proposed method has obvious advantages in practical bridge engineering.

Because sensors often appear the phenomenon of value drift, it is recommended not to start the early-warning when the single sample value exceeds the dynamic threshold. In the practical application process, it is essential to establish a multi – level warning mechanism. When a single monitoring sensor exceeds the threshold value at a single moment, no warning is issued. When a single monitoring sensor exceeds the threshold for several consecutive moments, the first – level warning is activated, and the original data is determined by other technical means. When multiple monitoring sensors exceed the threshold for several consecutive moments, the second – level warning is activated. When the second – level warning is triggered, engineers need to further inspect the bridge’s health and propose maintenance measures.

### 3.5. Permissions and approvals

For on-site data collection, permission to access the bridge and install sensors was approved by Jinan Urban Construction Group Co., Ltd. As the bridge contractors, they supervise the installation and commissioning of the bridge health monitoring system, and their approval ensures compliance with local regulations for infrastructure monitoring activities. Harbin Institute of Technology is responsible for the construction of the bridge health monitoring system, bridge structural safety analysis, and scientific research, and thus has the authority to utilize bridge monitoring data to conduct research and publish related research findings. No ethical or animal/human subject approvals were needed, as the study focuses solely on structural data without involving human participants or protected environments.

## 4. Conclusions

The structural response of bridges comprehensively manifests various coupling effects. Traditional methods frequently fail to capture the long-term trends in structural monitoring data. Consequently, it is arduous to effectively identify and issue warnings for abnormal monitoring data of bridge structures under the influence of complex coupled loads. In this paper, a dynamic early-warning method for bridge structural safety based on data reconstruction and depth prediction is proposed. This method is capable of accurately predicting the long-term monitoring data of bridge structures, thereby enabling real-time warning of abnormal data. The main conclusions are summarized as follows:

(1) The multi-dimensional monitoring data of the same bridge type are decomposed and reconstructed according to the contribution rates of influencing factors. Through the establishment of a deep learning architecture grounded in a Long Short-Term Memory (LSTM) network, the temporal correlation characteristics of each feature space within the structural monitoring data are effectively extracted. This approach allows for the formulation of a monitoring data prediction model, which facilitates dynamic early warning regarding bridge structural safety.
(2) Validation conducted on an actual bridge shows that the Root Mean Squared Error (RMSE) between the predicted and measured strain data after de-normalization is within 3 με, significantly exceeding the prediction accuracy of the SARIMA and SVM algorithms.
(3) Based on the established data prediction model, a dynamic early-warning method for bridge structural safety, driven by both predicted and measured data, is put forward. This method calculates the upper and lower limit thresholds for abnormal monitoring data more accurately, enhancing the early-warning accuracy and decreasing the probability of misjudgment.
(4) By incorporating simulated damage with an average value of 10 με and a variance of 0.01 (simulating data drift caused by concrete cracks, spalling, and wet joint damage) into the measured strain monitoring data, the proposed method effectively signals data anomalies.
